# Stathmin-like 4 is critical for the maintenance of neural progenitor cells in dorsal midbrain of zebrafish larvae

**DOI:** 10.1038/srep36188

**Published:** 2016-11-07

**Authors:** Meng-Ju Lin, Shyh-Jye Lee

**Affiliations:** 1 Department of Life Science, National Taiwan University, Taipei, Taiwan; 2 Research Center for Developmental Biology and Regenerative Medicine, National Taiwan University, Taipei, Taiwan; 3 Center for Systems Biology, National Taiwan University, Taipei, Taiwan; 4 Center for Biotechnology, National Taiwan University, 1 Roosevelt Rd., Sec., 4, Taipei, Taiwan

## Abstract

A delicate balance between proliferating and differentiating signals is necessary to ensure proper growth and neuronal specification. By studying the developing zebrafish brain, we observed a specific and dynamic expression of a microtubule destabilizer gene, *stathmin-like 4* (*stmn4*), in the dorsal midbrain region. The expression of *stmn4* was mutually exclusive to a pan-neuronal marker, *elavl3* that indicates its role in regulating neurogenesis. We showed the knockdown or overexpression of *stmn4* resulted in premature neuronal differentiation in dorsal midbrain. We also generated *stmn4* maternal-zygotic knockout zebrafish by the CRISPR/Cas9 system. Unexpectedly, only less than 10% of *stmn4* mutants showed similar phenotypes observed in that of *stmn4* morphants. It might be due to the complementation of the increased *stmn1b* expression observed in *stmn4* mutants. In addition, time-lapse recordings revealed the changes in cellular proliferation and differentiation in *stmn4* morphants. *Stmn4* morphants displayed a longer G_2_ phase that could be rescued by Cdc25a. Furthermore, the inhibition of Wnt could reduce s*tmn4* transcripts. These results suggest that the Wnt-mediated Stmn4 homeostasis is crucial for preventing dorsal midbrain from premature differentiation via the G_2_ phase control during the neural keel stage.

During embryonic development, cells proliferate and differentiate to assume different lineages needed for a mature organism. Precise controls on transitions between proliferation and differentiation are essential for proper patterning within compartments, in particular brain development. However, the regulatory mechanisms still remain largely unexplored. Zones of delayed neurogenesis are those areas confined and conspicuous during neurogenesis in vertebrates^1^,^2^. Among this, neuron-free midbrain-hindbrain boundary (MHB) or intervening zones is crucial for patterning and growth of midbrain-hindbrain^3^,^4^. Mutual interactions between Wnts and fibroblast growth factors (Fgfs) originated from the MHB are proposed to control patterning, cell fate decision and proliferation^5^,^6^,^7^. The Fgf gradient thus induces the expression of a hairy gene *her5* which prevents neuron differentiation invading from anterior brain via cell cycle regulation^8^ and inhibition of proneural gene expressions^9^. Although we know some of the core molecular switches mediating cell cycle exit during neuronal differentiation^10^ yet how the altered mitosis could direct cell fate determination is still far from clear.

Microtubules are the basal components of mitotic spindles that drive cell division during cell cycle progression. They are highly dynamic and are constantly switching between growth and shrinkage phases^11^. The microtubule cytoskeleton is also vital during neurogenesis by mediating neuronal migration or neurite growth. It thus implies that microtubule dynamics may be involved in maintaining the neural progenitor cell pools presumably through their associated proteins. Diverse microtubule-associated proteins, including stathmin family phosphoproteins, assist in particular neurogenesis phase by mediating microtubule dynamicity. Stathmin family genes, comprised of stathmin, SCG10, SCLIP, and RB3 with its splice variants RB3’ and RB3”, share a highly conserved stathmin-like domain (SLD), which binds with two tubulin heterodimers to form a “T2S” complex^12^. Mostly by the sequestering of free tubulins, stathmins are regarded as microtubule destabilizers and intracellular signaling relay centers. In addition to stathmin, other stathmin member proteins possess an additional N-terminal extension. With palmitoylations onto two conserved cysteine residues and conserved sequence AYKEMKEL in domain A of the N-terminal extension, stathmin phosphoproteins could be tethered to Golgi membranes or other vesicle-like structures^13^.

Even with differential expressions, all four stathmins are highly enriched in the nervous system not only during development but also adult stages. Through modulating neurite outgrowth or axon branching, stathmins are recognized as neuronal differentiation hallmarks. In addition, cellular migration involving tissue patterning is associated to stathmins. Stathmins have also been reported to influence neuronal proliferation^14^.

We have previously demonstrated distinct expression of 4 of 7 stathmin genes in the central nervous system during development in zebrafish embryos^15^. Among them, *stathmin-like 4* (*stmn4*) called our attention due to its restricted expression within the neuron-free midbrain during a critical period of brain development. We demonstrate here that the homeostasis of *stmn4* expression is indispensable for neuron progenitor cell pool maintenance in the dorsal midbrain. Either overdose or reduction of *stmn4* expression led to premature appearance of post-mitotic neurons. Although the premature differentiation was only observed in a small portion of *stmn4* mutants generated, it might be due to the complementation of increased *stmn1b* expression. With stochastically labeling, single-cell resolution analysis further confirmed the link between Stmn4 dynamics and proliferation within intervening zones. A longer G_2_ phase observed in *stmn4*-deficient embryos appears to be critical to drive neuron progenitors exit from cell cycle and subsequent neurogenesis. Thus, we conclude that Stmn4 is vital for preventing premature neurogenesis to keep progenitor cell pools within dorsal midbrain during early brain development.

## Results

### *stmn4* is highly expressed before neuronal differentiation in dorsal midbrain

**Figure 1:**
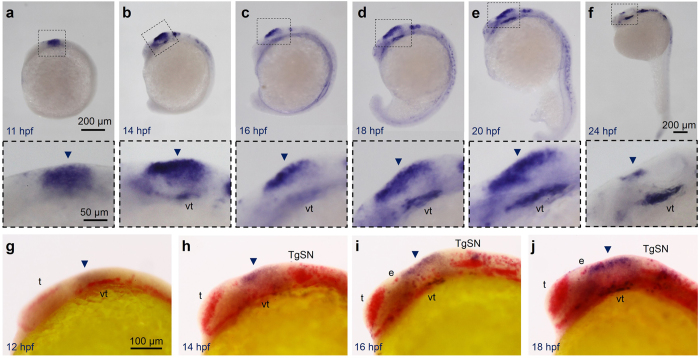
*Stmn4* prohibited *elavl3* expression in the dorsal midbrain during early embryogenesis. (**a**–**f**) Representative photographs of whole-mount *in situ* hybridization against *stmn4* in zebrafish embryos at designated stages are displayed. *stmn4* were highly expressed in the midbrain regions (boxed). A magnified photograph of the boxed regions is shown beneath each panel. (**g**–**j**) Representative photographs of double whole-mount *in situ* hybridization against *stmn4* (purple) and *elavl3* (red) at designated stages are presented. Arrowheads point to the dorsal midbrain. Scale bars are shown in the panels (**a**,**g**) on the right for each row unless specified in (**f**). Embryonic stages are shown in hours post fertilization (hpf) at the lower left corner in each panel. e: epiphysis; t: telencephalon; TgSN: trigeminal ganglion sensory neurons; vt: ventral tegmentum.

To investigate the role of Stmn4 in dorsal midbrain development, we performed whole-mount *in situ* hybridization (WISH) to examine its expression patterns in the developing brain of zebrafish embryos from 11–24 h post fertilization (hpf). *stmn4* was not expressed until 11 hpf (Fig. 1a, data not shown for those embryos before 11 hpf). It first appeared at the dorsal midbrain region and then ventral tegmentum (Fig. 1b–e) until 20 hpf. The expression of *stmn4* in the dorsal midbrain decreased abruptly, but persisted in the ventral tegmentum at 24 hpf (Fig. 1f). The *stmn4* transcripts were also found in spinal neurons (Fig. 1b–f). Since neurogenesis of dorsal midbrain occurs late in embryogenesis, we hypothesized that Stmn4 may be inhibitory to neurogenesis. To test the hypothesis, we performed double WISH against *stmn4* and *ELAV like neuron-specific RNA binding protein 3* (*elavl3*). The *elavl3* is the current annotated name for *huc*, a pan-neuronal marker, and will be used hereafter. Notable mature neurons first arouse in telencephalon and ventral tegmentum at 12 hpf (Fig. 1g), expanded into trigeminal sensory neurons (TgSNs) at 14 hpf (Fig. 1h) and then epiphysis (e) at 16 hpf (Fig. 1i). In the dorsal midbrain, the *stmn4* expression domain was distinct from the surrounding *elavl3* expression (Fig. 1g–j, arrowheads). It suggested that *stmn4* may prohibit neuronal differentiation to maintain the neuronal progenitor pools in the dorsal midbrain.

### Characterization of Stmn4

**Figure 2:**
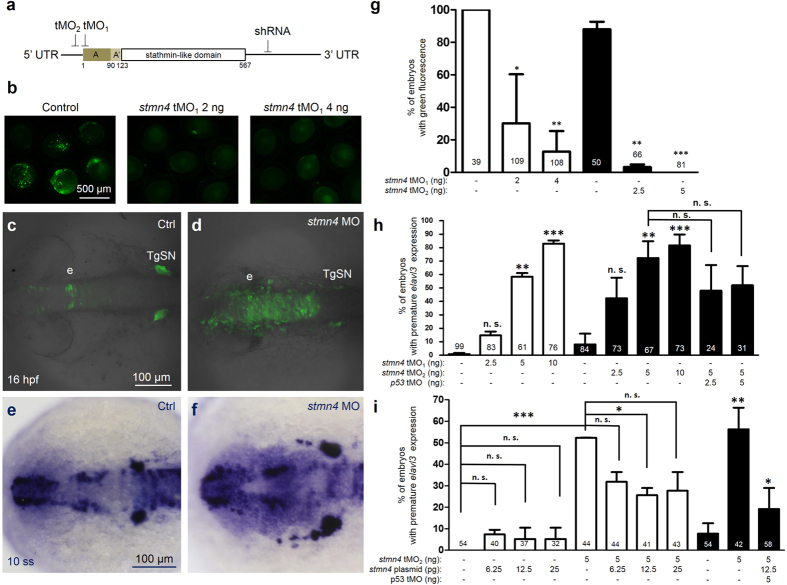
*stmn4* knockdown resulted in premature expression of *elavl3* in zebrafish embryos. (**a**) Target sites are indicated for two antisense translational blocking morpholino oligo nucleotides (tMO_1_ and tMO_2_) and one small hairpin RNA (shRNA) at the start codon, 5′ and 3′ untranslated region (UTR), respectively. (**b**) Embryos were injected with 200 pg of PCS2+ vector with an insertion of a fragment of *stmn4* containing both tMO_1_ and tMO_2_ binding sites as indicated in (**a**) without (control) or with tMO_1_ or tMO_2_ (*stmn4* MO) as indicated, examined and photographed at the bud stage. The percentages of embryos with green fluorescence were calculated and compared to the control groups as shown in (**g**). Tg(*Huc:kaede*) embryos were injected without (**c**, Ctrl) or with different amounts of *stmn4* tMO_1_ or tMO_2_ (**d**, *stmn4* MO)*, P53* MO and *stmn4* expression vectors, cultured to 16 hpf and examined under confocal microscopy. A stack of 10 layers (2.5 μm for one layers) from the top of midbrain were combined and shown. (**e**–**i**) Wild type embryos were treated as described previously, cultured to 10-somite stage, fixed and subjected to WISH against *elavl3*. Representative photographs are presented (**e**,**f**). Scale bars are only shown on the left panel in a row. A premature elevation of *elavl3* expression was observed in embryos injected with *stmn4* MO (**f**) compared that of control. The percentages of embryos with elevated *elavl3* expression in the dorsal midbrain were calculated and shown in (**g**–**i**). Photographs are at the same scale in each row and scale bars are only shown in the left panels. Experiments were repeated three times. Data was analyzed by one-way ANOVA and presented as mean +/−s.e.m. *P < 0.05, **P < 0.005, ***P < 0.0005. The numbers of embryos used are shown at the top or bottom of each bar.

All stathmin family genes possess a SLD and an additional N-terminal domain except for *stathmin1b* (*stmn1b*) (Fig. 2a). The Stmn4 contains two conserved cysteine residues, C20 and C22, which can be palmitoylated to enhance the recruitment of Stmn4 to Golgi apparatus. We mutated the C20 and C22 residues of Stmn4 to alanine and found that the recruitment of Stmn4 to Golgi apparatus is significantly reduced in Hela cells (Fig. S1). In addition, the Stmn4 SLD has the first three conserved serine residues, S59, S71 and S81, but does not have the fourth serine reside found in their mammalian homologues (Fig. S2a). Phosphorylations of these serine residues hamper the microtubule depolymerizing ability of stathmins^16^. Thus, we changed all these serine residues of Stmn4 to alanine to make it a non-phosphorylatable constitutively active form denoted as CA-Stmn4, which presumably has a higher microtubule depolymerizing activity. The CA-Stmn4 plasmids were injected into 1-cell stage zebrafish embryos, subjected to immunohistochemistry against histidine and alpha-tubulin to reveal Stmn4 and microtubule, respectively, and examined under confocal microscopy. As shown in Fig. S3(b,c), the percentage of polymerized microtubules in each blastomere appears to be less and negatively correlated to the expression of *ca-stmn4*. On the other hand, the percentage of depolymerized microtubules in each blastomere is higher and positively correlated to the expression of *ca-stmn4* (Fig. S3b,d). The overexpression of *ca-stmn4* driven by a CMV promoter interferes gastrulation, a process mediated by microtubule dynamics^17^. It further validates the higher microtubule destabilizing activity of CA-Stmn4 (Fig. S2b). Collectively, these results clearly demonstrated that the zebrafish Stmn4 contains its basic characteristics and is functionally similar to their mammalian homologues.

### Knockdown of *stmn4* causes precocious neuronal differentiation in dorsal midbrain

To understand whether Stmn4 is involved in maintenance of neural progenitor state in the dorsal midbrain, two antisense translation-blocking morpholino oligonucleotides (MOs) against *stmn4* were designed to target start codon (tMO_1_) and 5′ untranslated region (UTR, tMO_2_) as indicated in Fig. 2a. We examined the efficiency of MO by co-injecting the pCS2+ XLT-GFP vector with an insertion of *stmn4* fragment containing both MO target sites without or with designated dosages of MOs. The control group showed mosaic GFP fluorescence in most embryos that was in clear contrast to the notably reduced fluorescence in MO-injected embryos, which will be referred as morphants hereafter (Fig. 2b). The percentage of embryos expressing GFP was dose-dependently reduced in morphants of both MOs (Fig. 2g). Both tMO_1_ and tMO_2_ morphants were grossly normal throughout gastrulation (data not shown).

To address the regulation of neurogenesis by Stmn4, we used a transgenic *Tg(HuC:Kaede)* line which expressing green Kaede fluorescence in differentiated neurons^18^. At 16 hpf, Kaede-labeled differentiated neurons were mainly found in epiphysis and trigeminal ganglion neurons (TgSNs) but not in the dorsal midbrain (area between epiphysis and TgSN) of control embryos (Fig. 2c). In contrast, differentiated neurons appeared in dorsal midbrain in up to 80% of *stmn4* morphants (Fig. 2d,h). Similar results were obtained by WISH analysis against *elavl3* (Fig. 2e,f). The percentage of embryos with precocious *elavl3* expression was MO dose-dependent in both *stmn4* morphants (Fig. 2h). tMO_2_ appeared to be more effective than tMO_1_, so it was used for the rest of experiments.

To figure out whether the precocious *elavl3* expression observed in *stmn4* morphants was specifically due to the depletion of endogenous Stmn4, a plasmid containing the coding region sequence of *stmn4* was co-injected with 5′ UTR targeting tMO_2_ into embryos and found significant (P < 0.05) but partial rescue of phenotypes. In addition, the injection of *stmn4* plasmids had no significant effect on precocious *elavl3* expression at the dosages tested (Fig. 2i). We also tested whether the effect of *stmn4* MOs might be caused by non-specific activation of P53^19^. The *stmn4* MO-induced expression of *elavl3* was partially alleviated by co-injecting *p53* MO but not significant (Fig. 2h). Taken together, these results indicated the depletion of Stmn4 could specifically induce the precocious *elavl3* expression in dorsal midbrain during early development.

### Targeted mutation of *stmn4* causes mild precocious neuronal differentiation in dorsal midbrain and the increase in *stmn1b* expression

**Figure 3:**
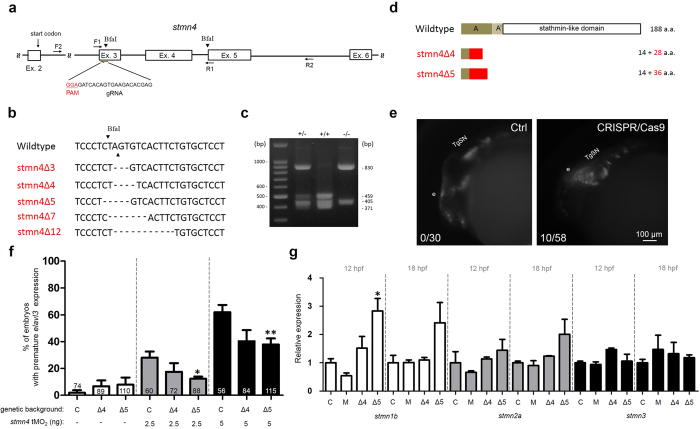
Targeted mutation of *stmn4* increased *stmn1b* expression and caused mild precocious neuronal differentiation in dorsal midbrain. (**a**) A partial genomic structure of *stmn4* shows the Exon 3 targeting gRNA site and sequence (PAM site in red). The two BfaI sites (arrowheads) and the sites complementary to the forward (F) and the reverse (R) primers (arrows) used for cloning for sequencing and restriction digestion assay are shown. (**b**) The sequence flanking the deletion site *stmn4* mutants (stmn4Δ3-12) are shown. The deleted nucleotides are denoted by dashes. The cut sites of Bfa1 are indicated by arrowheads. (**c**) Restriction digestion by BfaI. The genomic DNAs from tail fins of wild type (+/+), heterozygous (+/−) or homozygous (−/−) stmn4Δ5 were isolated to be used as templates for PCR using the forward and reverse primers indicated in (**a**). The amplicons were digested and run in agarose gels and stained. A representative gel image is shown. The sizes in bp for selected DNA marker bands and digested fragments are shown. (**d**) Two *stmn4* mutant alleles (stmn4Δ4 and stmn4Δ5) have a premature stop codons that resulted in truncated Stmn4 proteins as shown lacking all known functional domains. (**e**) Partial activation of precocious *elavl3* expression in dorsal midbrain of F0 *Tg*(*Huc:Kaede*) embryos by *stmn4* CRISPR/Cas9 compared to that of control embryos (Ctrl). (**f**) The % of premature *elavl3* expression was determined in wildtype (C), stmn4Δ4 (Δ4) and stmn4Δ5 (Δ5) embryos-injected with indicated concentration of *stmn4* tMO_2_. (**g**) Relative expression of *stmn1b*, *stmn2a* and *stmn3* were measured in wildtype (C), morphant (M), stmn4Δ4 (Δ4) and stmn4Δ5 (Δ5) embryos at 12 and 18 hpf by qPCR. Data was analyzed by one-way ANOVA and presented as mean +/− s.e.m. *P < 0.05, **P < 0.005, ***P < 0.0005.

To further confirm the function of Stmn4 genetically, we generated *stmn4*^−/−^ mutants in the transgenic *Tg(HuC:Kaede)* background using the Clustered regularly interspaced short palindromic repeats (CRISPR)/Cas9-mediated approach^20^. We designed several guide RNAs (gRNAs) targeting different sites on the *stmn4* gene. In brief, different gRNAs were separately co-injected with Cas9 mRNAs into 1-cell stage embryos. Survived embryos were raised to adulthood and then crossed to wildtype fish to obtain F1 embryos. The F1 embryos were raised to two months old and subjected to the Sanger sequencing analysis. Zebrafish fin genomic DNAs were used as templates for polymerase chain reaction (PCR) using a pair of primers (F1 and R1 as denoted in Fig. 3a). The PCR products were then sequenced to identify mutant founders. One of the gRNAs targeting the Exon 3 (sequence shown in Fig. 3a) effectively created deletions in 5 different mutant alleles (stmn4Δ3-12) of the *stmn4* gene (Fig. 3b). The identified F1 founders were then in-crossed within each allele to acquire F2 generation. To identify homozygous mutants in F2 fish, we found a BfaI site (CTAG) within all the deletions and a second BfaI site at the beginning of Exon 5. The BfaI sites were thus useful for the rapid screening of mutant alleles by restriction digestion (Fig. 3a). We then raised the identified mutant founders to two month old, collected their fin genomic DNAs and subjected to restriction digestion assay to separate wildtype, heterozygous and homozygous *stmn4* mutant fish (Fig. 3c). To perform the restriction digestion assay, zebrafish fin genomic DNAs were used as templates for PCR using a pair of primers (F2 and R2 as denoted in Fig. 3a) to give rise a 1235 bp PCR product. Taking the stmn4Δ5 allele as an example, the PCR product was cleaved by BfaI into three fragments (459, 405 and 371 bp) in samples from wildtype embryos. In clear contrast, two fragments (830 and 405 bp) were present in samples from homozygous mutants (−/−) due to the loss of first BfaI site. However, due to the presence of both wildtype and mutant PCR products, lower amounts of 459 and 371 bp fragments also existed in samples from heterozygous mutants (Fig. 3c). We also confirmed the validity of the restriction digestion by sequencing randomly selected 1235-bp PCR products (data not shown). Two of those mutant alleles containing 4 (stmn4Δ4) or 5 bp deletion (stmn4Δ5) near the protospacer adjacent motif (PAM) site caused premature stop codons and presumably should result in a truncated Stmn4 with 14 N-terminal and 28 or 36 missense (in red) amino acids, respectively, as shown in Fig. 3d. Both alleles lacked all Stmn4 functional domains and their F2 homozygous mutants were raised to adulthood. The F2 homozygous mutants of stmn4Δ4 and stmn4Δ5 alleles were in-crossed to produce maternal zygotic mutant embryos. Although mutants were generated in the transgenic *Tg(HuC:Kaede)* background, however the fluorescence intensity of Kaede was too low after repeated crossing (data not shown). Therefore, we used WISH against *elavl3* to examine the premature neurogenesis in the dorsal midbrain. Unexpectedly, most of the mutant embryos from both alleles developed normally. Only 5 of 89 (6.7%) in stmn4Δ4 mutant embryos and 7 of 110 (7.8%) stmn4Δ5 mutant embryos showed premature *elavl3* expression in dorsal midbrain (Fig. 3f). The lack of phenotype in most embryos from the *stmn4* mutant alleles implied possible artifacts observed in the previous MO-based studies. To address this issue, we tested the response of embryos from both mutant alleles to the *stmn4* tMO_2_ and found that the *stmn4* tMO_2_-induced premature *elavl3* expression in dorsal midbrain was notably and significantly lower in stmn4Δ4 and stmn4Δ5 mutant embryos, respectively, compared to that in wildtype embryos (Fig. 3f). This indicated that the observed premature *elavl3* expression in dorsal midbrain was specifically induced by *stmn4* tMO_2_. Furthermore, the F0 embryos-injected with the Exon 3 gRNAs revealed brain malformation and massive death similar to that observed in *stmn4* morphants (data not shown). We also observed lower but notable precocious *elavl3* expression in dorsal midbrain in 10 of 58 Exon 3 gRNAs/Cas9-treated embryos (Fig. 3e). These results suggested possible functional complementation by other stahmin isoforms or gene compensation^21^ that might mask the phenotypes resulted from the loss of *stmn4*. Thus, we examined the gene expression of other stathmin genes in stmn4Δ5^−/−^ mutants at 12 and 18 hpf and found that the expression of *stmn1b* but not other stathmin genes was significantly (P < 0.05) and notably elevated at 12 and 18 hpf, respectively (Fig. 3g).

### Stmn4 homeostasis is essential for neural progenitor cell maintenance in dorsal midbrain

**Figure 4:**
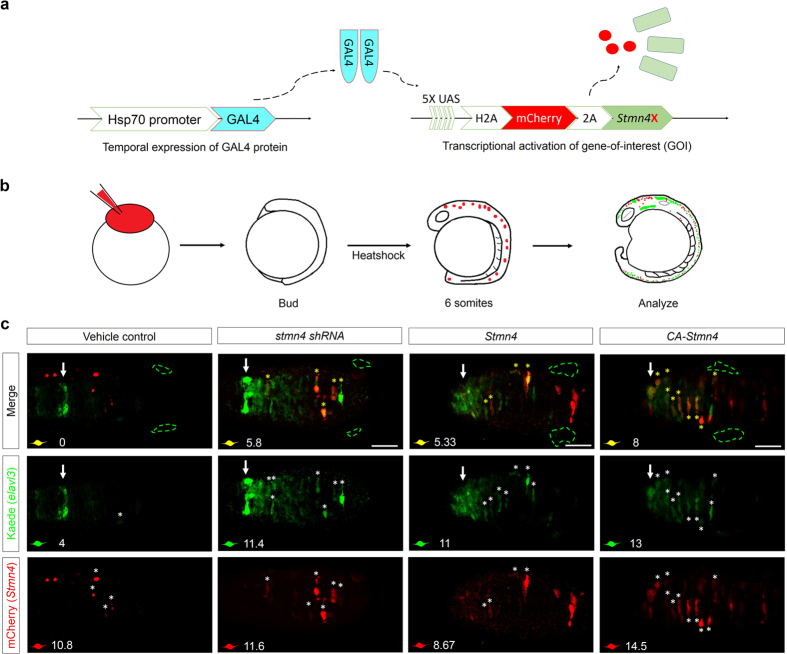
Overexpression of CA-*Stmn4* or depletion of *stmn4* by shRNA induced *elavl3* precocious expression in dorsal midbrain. (**a**) Conditional gene expression system was introduced by combining Gal4-UAS system with heat shock 70 (hsp70) promoter. After heat shock, upstream activation sequence (UAS) elements will be recognized by Gal4 protein dimers (indicated by light blue) and then activate transcription of downstream genes including nuclear H2A-mCherry in red and *Stmn4* intervening with a viral 2A peptide. (**b**) Flow chart showing the conditional expression system. We heat-shocked the *Tg(HuC:Kaede)* embryos injected with both plasmids described above until bud stage. After two hours of induction, embryos were analyzed until 16 hpf for *elavl3* expression revealed by Kaede (green). (**c**) Dorsal view of midbrains between epiphysis (indicated by white arrows) and (TgSNs, circled by green dashed lines) are shown. Within one embryo, average number of precocious *elavl3* (demonstrated by Kaede, green) expressing cells colocalized with (yellow) or without overexpressions of *stmn4* either with mutations or its corresponding shRNA^*stmn4_614*^ (indicated by mCherry, red) in dorsal midbrain, are shown on the left-bottom corner of each panel. Scale bars are 50 μm.

To further confirm the *stmn4* MO-induced precocious *elavl3* expression in dorsal midbrain, we utilized shRNA targeting the 3′ UTR of *stmn4* (Fig. 2a) by employing the UAS-miR-30 backbone in combination with the Gal4-UAS system. We used two vectors for the Gal4-UAS induction system. One vector was constructed with a heat shock protein 70 (hsp70) promotor and a Gal4 gene^22^. Another vector contains 5X UAS elements, a tdTomato and a *stmn4* shRNA which is situated at the 3′ UTR of tdTomato (modified from a previously reported vector^23^). We injected both vectors into 1-cell embryos, cultured to the bud stage and then heat shocked for 2 h to avoid the possible interference it may cause during early embryogenesis (Fig. 4b). The hsp70 promoter can be activated by heat shock to drive *gal4* expression. Gal4 protein can then bind to the 5XUAS elements to trigger the expression of *tdTomato* and *stmn4* shRNA. The *stmn4* shRNA of 21 nucleotides was designed to target nucleotide 614 at the 3′ UTR of *stmn4* (Fig. 2a). The expression of shRNA^*stmn4_614*^ effectively reduced the expression of *stmn4* to 0.25% in 1-day-old embryos compared to that in control embryos by quantitative PCR (qPCR) analysis. All embryos were fixed at 16 hpf and subjected to immunohistochemistry probed with anti-Kaede and mCherry antibodies. We used the anti-mCherry antibody because it can cross recognize tdTomato. The dorsal mid brain area was depicted within the region between the epiphysis (arrow) and the TgSNs (green dashed circles) as shown in the first row of Fig. 4c. We examined the dorsal mid brain area under confocal microscopy to screen 10 slices (25 μm thick in total) and to see whether the expression of shRNA^*stmn4_614*^ (as indicated by the expression of tdTomato in red) can induce the precocious *elavl3* expression (as indicated by the expression of Kaede in green). In three trials, we observed the stacked images from all slices and found 63.2% of 37 embryos exhibited precocious *elavl3* expression in dorsal midbrain that confirmed the phenotypes of *stmn4* morphants. We further counted fluorescent cells within dorsal midbrain. However, due to the respective background expression of tdTomato and Kaede, only those cells with respective fluorescence above a certain threshold was counted as positive cells (denoted with “*”). We observed an average of 10.8 tdTomato- and 4 Elavl3-positive cells in each vehicle (the same construct without stathmin) control embryos. In contrast, an average of 11.6 tdTomato- and 11.4 Elavl3-positive cells were found in each shRNA^*stmn4_614*^-treated embryos. Among those fluorescent cells, an average of 5.8 cells expressed both signals (Fig. 4c). These results clearly demonstrated at the cellular level that the loss of Stmn4 induces precocious neural differentiation.

On the other hand, we also performed gain of function assay of Stmn4 by overexpressing wildtype *stmn4* or *ca-stmn4* to test their effects on precocious *elavl3* expression in dorsal midbrain. We employed a similar strategy by using the same hsp70-driven Gal4 vector and a vector with 5X UAS, H2A-mCherry, 2A peptide and wildtype Stmn4 or CA-Stmn4 for conditional expression (Fig. 4a). The 2A peptide was used to separate mCherry and Stmn4^24^. Following similar protocols as used in the *stmn4* shRNA experiments (Fig. 4b), we observed an average of (1) 8.67 mCherry- (white asterisks), 11 Elavl3-positive (white asterisks) and 5.33 double-positive cells (yellow asterisks) were found in each *stmn4*-treated embryos. (2) 14.5 mCherry-, 13 Elavl3-positive and 8 double-positive cells were found in each *ca stmn4*-treated embryos (Fig. 4c). In three trials, we observed from the stacked images that 54.8% of 36 *stmn4*- and 68.9% of 32 *ca-stmn4*-overexppressing embryos exhibited precocious *elavl3* expression in dorsal midbrain. Collectively, these results demonstrated that either the loss or gain of Stmn4 can induce precocious neural differentiation and suggested an essential role of Stmn4 homeostasis in the maintenance of neuronal progenitor cells in the dorsal midbrain of zebrafish embryos.

### Stmn4-mediated precocious neuronal differentiation occurs during the neural keel stage

**Figure 5:**
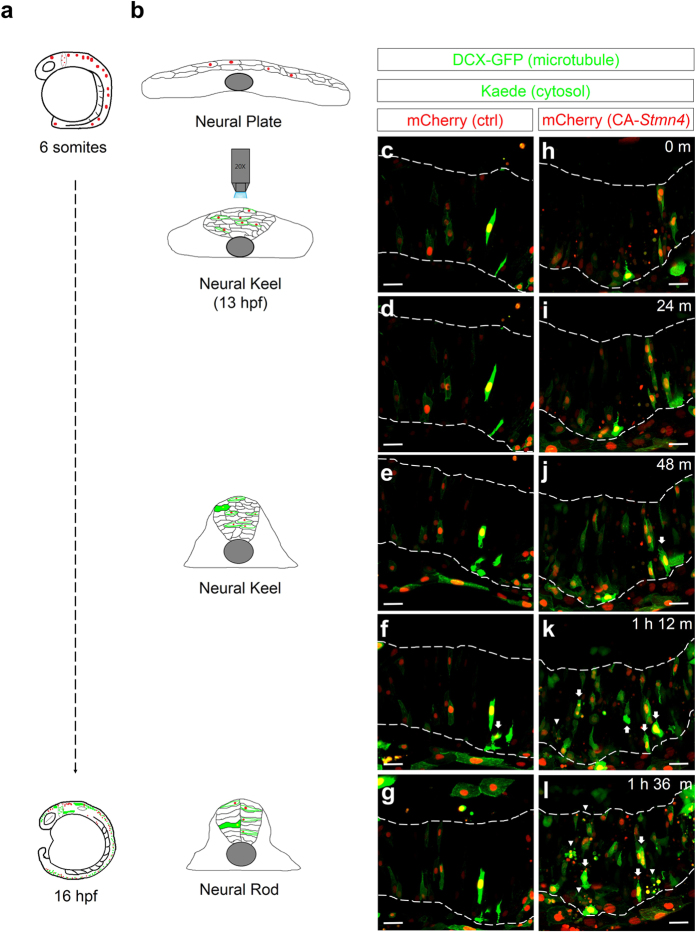
Overexpression of CA-*Stmn4* induced precocious expression of *elavl3* in neuronal precursor cells during neural keel stage. Time-lapse movies were conducted 6 minute per frame around the neural keel stage (13 hpf) after heat shock (6-somite stage). During neurulation (**b**), neural progenitor cells converge and migrate to the dorsal midline to establish neural rod (16 hpf) from a flattened neural plate (bud stage). Here, microtubules are revealed by doublecortin-GFP fusion protein (DCX-GFP, green, indicates thread-like microtubules structure depicting the cell shape). HuC:Kaede (green) expresses within cytoplasm. Compared to control embryo (**c**–**g**), more cells with *elavl3* expression (arrows) and more apoptotic cells (arrowheads) appeared in embryo overexpressing CA-Stmn4 (mCherry, red) during the neural keel stage (**h**–**l**), characterized by a disorganized and thickening tissue (**b**). Corresponding time points are shown on the upper-right corner. Boundaries of neural tissues are disclosed by a pair of dash lines. All snapshots shown in dorsal side and anterior to the left. Scale bars equal 25 μm for all.

To further investigate the progression of precocious neuronal differentiation in *ca-stmn4*-overexpressing cells, we conducted time-lapse recordings shortly after the heat shock at the 6-somite stage to 16 hpf in the dorsal midbrain of developing zebrafish embryos (Supplementary Videos 1 and 2). Snapshots of selected frames as designated are presented in Fig. 5c–l. In addition to two vectors used, the Medusa vector M2 containing doublecortin-GFP (DCX-GFP) was introduced to monitor the microtubule dynamics^25^. At the beginning, neural progenitor cells, stochastically highlighted by nucleus in red and microtubules in green (thread-like structure), were interdigitated with each other between the boundaries of neural tissues (Fig. 5c,h; dashed lines), forming a pseudostratified layer, which is called the “neural keel” stage (Fig. 5b; middle). Those cells oscillated between the boundaries and migrated medially (convergence) with final C-divisions (cross-midline division)^26^ giving the mirror-symmetric daughter cells and to form the midline as the key feature of the “neural rod” stage (Fig. 5b; bottom). At 16 hpf, few mature neurons (arrows, showing strong Elavl3 green fluorescence in cytosol) emerged in wildtype embryos (Supplementary Video 1; Fig. 5c–g; arrows). In contrast, differentiated neurons (arrows) appeared promptly at 14 hpf (neural keel stage) after heat shock in the embryos-injected with the *ca-stmn4* plasmids (Supplementary Video 2; Fig. 5h–l). Typically, neurogenesis develops through asymmetric division which generates one daughter neuronal cell and the other remains as a progenitor cell. However, the vast majority of neuronal differentiation arose before cell division (71.4%), which suggested that the disturbance of Stmn4 homeostasis induces neuronal differentiation via an asymmetric division-independent mechanism. Besides, some progenitor cells underwent morphological changes with fragmented microtubules and eventually were apoptotic regardless of precocious *elavl3* expression (Supplementary Video 2; Fig. 5h–l; arrowheads). It thus implies that overdose of CA-Stmn4 could disrupt cell viability through interruption of microtubule dynamics. Taken together, both precocious neurogenesis and apoptosis occurred during the neural keel stage in those *ca-stmn4*-overexpressing embryos.

### Knockdown of *stmn4* prolongs G_2_ phase to trigger precocious neuronal differentiation in dorsal midbrain

**Figure 6:**
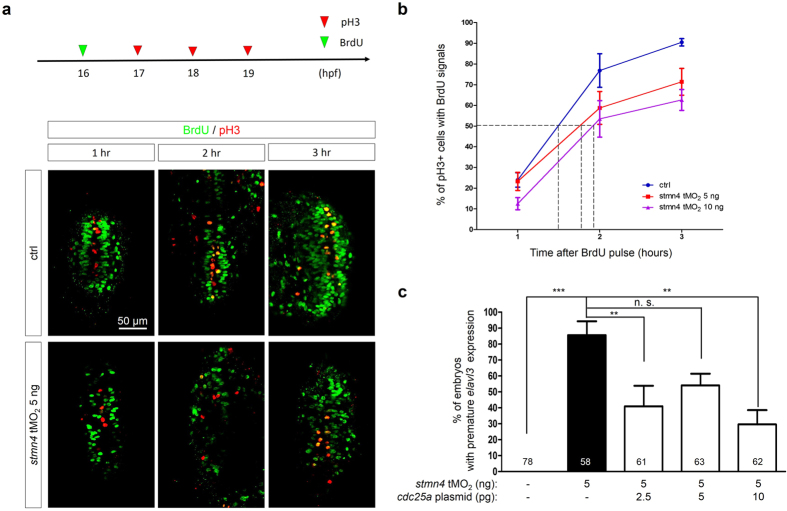
Deficiency of *stmn4* prolonged the G_2_ phase in a Cdc25a-dependent manner. (**a**) 1-cell stage embryos were injected without (Ctrl) or with 5 and 10 ng *stmn4* tMO_2_. A 15-min BrdU pulse (green arrowhead) was then given to embryos at 16 hpf. Part of embryos were fixed at an hour intervals from 17–19 hpf and subjected to double immunochemistry against α-BrdU (green) and α-pH3 (red). Fixed embryos were examined and photographed under confocal microscopy. Representative photos are presented for each treatment at designated times post the BrdU pulse. All images are shown in dorsal views with anterior to the top. Percentages of pH3+ and BrdU+ cells (yellow cells) within the pH3+ cells (yellow and red cells) were calculated as shown in (**b**) and analyzed by one-way ANOVA. (**c**) 1-cell stage Tg(*Huc:Kaede*) embryos were injected without or with 5 ng *stmn4* tMO_2_ and indicated amounts of *cdc25a* plasmid. The percentages of embryos with premature *elavl3* expression were examined and presented as described in Fig. 2.

To understand the mechanism underlying the early post-mitotic neuron cells appearance in *stmn4* morphants, we investigated whether the deficiency of Stmn4 may alter cell cycle which is tightly associated with neuronal differentiation. We analyzed the length of G_2_ phase by using the percentage of labeled mitosis (PLM) method^27^,^28^. In brief, embryos were treated with short BrdU pulses at 16 hpf and then fixed sequentially at 1 to 3 h after treatments (Fig. 6a). Fixed embryos were subjected to immunohistochemistry probed with anti-BrdU and anti-phospho-Histone H3 (pH3) antibodies to reveal cells in S phase (green) and M phase (red). Then, the length of G_2_ phase was measured by the duration between the S and M phases (Fig. 6b,c). We noted that cell proliferation was obviously reduced in *stmn4* morphants (n = 8.4 with 5 ng MO; n = 7.3 with 10 ng MO) compared to that in wildtype embryos (n = 12.6) by counting the number of pH3-positive cells within the dorsal midbrain. The percentages of pH3/BrdU positive cells were notably lower in *stmn4* morphants compared to that in wildtype embryos. Extrapolating from the curves, we found that the average length of G_2_ phase (50% of BrdU positive cells with pH3 staining) was increased dose-dependently from 1 h and 29 min to almost 2 h. (Fig. 6b). The cell cycle is carefully administered by cyclins and cyclin-dependent kinases (CDKs). Among them, Cyclin B forms a complex with Cdk1 to guard the G_2_/M checkpoint through the regulatory phosphorylation cascade^29^. We found that precocious neuronal differentiation could be rescued by overexpressing *cdc25a*, an upstream regulatory element of CyclinB/Cdk1, in *stmn4* morphants (Fig. 6c). It suggested that the control of G_2_/M transition by Stmn4 is key to its maintenance of neuronal progenitor cells.

### Inhibition of Wnt signaling downregulates the expression of *stmn4*

**Figure 7:**
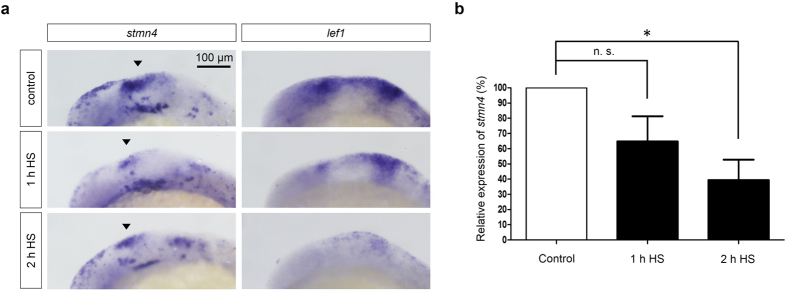
Block of Wnt signaling inhibited the expression of *stmn4*. *Tg(hsp70l:dkk1-GFP)* embryos were cultured to 10 hpf and then heat-shocked (HS) for 1 and 2 hr as indicated. Embryos were further cultured, fixed at 18 hpf and then subjected to WISH against s*tmn4* or *lef1* (**a**) or qPCR analysis against s*tmn4* using *actin* as an internal control (**b**). Data represents mean +/− s.e.m. and analyzed by one-way ANOVA (N = 5), *P < 0.05.

Canonical Wnt signaling is one of the key signal to mediate midbrain development by regulating proliferation and morphogenesis with a bi-modal control upon FGF activity^7^,^8^. To understand whether Wnt morphogens may regulate *stmn4*, we suppressed Wnt activity by applying transgenic fish *Tg(hsp70l:dkk1-GFP)*^*w32*^ to overexpress *dkk1*, a Wnt signaling inhibitor, and to examine the expression of *lef1* (a Wnt-activated downstream gene) and *stmn4* (Fig. 7a). WISH analysis revealed that the expression of *lef1* was reduced by *dkk1* expression induced by heat shock compared to that of control embryos. It demonstrated the effective inhibition by Wnt signaling. More importantly, we observed the notable reduction of *stmn4* expression in dorsal midbrain (Fig. 7a). This Wnt-dependent *stmn4* expression was also shown by qPCR analysis in RNA extract of whole embryos (Fig. 7b). This indicated the Wnt signaling may regulate *stmn4* expression at the transcriptional level.

## Discussion

Balanced transitions between proliferation and differentiation are keys to proper patterning during brain development. Their regulations within compartments are spatial and temporal-dependent that is even more complicated and still remains largely unknown. The brain isthmus in between midbrain and hindbrain is an organizer directing the growth and differentiation and is an excellent platform to study this unresolved issue. In this study, we demonstrate a novel role of a microtubule destabilizer Stmn4 in maintaining neural progenitor cell pools in dorsal midbrain during early brain development. We are the first to discover the adequate expression of *stmn4* is critical to prohibit the G_2_ phase of neural progenitors from lengthening that may cause early cell cycle exit and neurogenesis in the developing dorsal midbrain.

Stmn4 is less well studied comparing to other mammalian stathmins. Here, we first fully characterized the zebrafish Stmn4 owing features of tubulin sequestering and Golgi anchoring. However, since the cooperation of two subdomains within domain A of N-terminal extension controls the shuttling between Golgi apparatus and mitochondria^13^, whether palmitoylation-deficient Stmn4 could be recruited to mitochondria needs further investigation.

To study the *in vivo* function of Stmn4 in zebrafish, we demonstrated a possible role of Stmn4 in maintaining the neuronal progenitor pools by the perturbation of *stmn4* expression or activity resulted in precocious neural differentiation. However, we also observed inconsistent phenotypes between morphants and mutants as reported by other studies^21^,^30^. It implies possible off-targeting effects of *stmn4* MOs used. We revealed that the *stmn4* tMO_2_ was less effective in triggering precocious neural differentiation in *stmn4* maternal zygotic mutants that suggests the specific effects of *stmn4* MOs^21^. In addition, CRISPR/Cas9 also caused similar phenotypes in F0 embryos. Although at a lower penetrance, it could be attributed to the mosaic expression frequently observed by the transient expression in F0 embryos. Collectively, these results suggest possible cellular compensations due to the loss of Stmn4. Other stathmin family isoforms might be functional complementary to Stmn4. The loss of Stmn4 might also be compensated by other molecules sharing similar properties or acting downstream of mutated factors^21^ that is a common features in lower vertebrates^31^. We addressed these issues by demonstrating the upregulation of *stmn1b* in stmn4Δ5^−/−^ embryos. In addition, we also found that injection of *stmn1b* MO into stmn4Δ 5^−/−^ embryos could partially induce precocious expression of *elavl3* in dorsal midbrain (data not shown). It implicates that Stmn1b might complement the loss of Stmn4 to prevent premature neuronal differentiation in dorsal midbrain. Thus, double-knockout zebrafish of *stmn4 and stmn1b* are required for further studies. However, we still cannot rule out the possible involvements of other compensatory factors in regulating phenotypes of *stmn4* mutants.

It is difficult to resolve the controversy for the inconsistent phenotypes between morphants and targeted mutants. So one needs to be cautious not to over interpreting data form each side. Although the knockout approach appears to be a more clear-cut gene inactivation, but it is often complicated by gene complementation and compensation as discussed previously. The knockdown approaches although still have residual gene activity and presumably should have weaker impact comparing to the knockout approach, but cellular compensation is less likely to be an issue and it still allows to see the phenotypes due to gene deficiency. However, the nonspecific secondary effects of MOs should be of concern, so we have applied different controls as shown in Fig. 2 to exclude the possible MO artifacts. In conjunction with the shRNA and constitutively activated Stmn4 studies that both showed the induction of premature neuronal differentiation at the cellular level, it suggests that Stmn4 is a critical regulator to keep the cell in the dorsal midbrain in a progenitor status for regional specification during early brain development in zebrafish.

Stathmin as a negative regulator for neurogenesis appears to be contradictory to previous findings that stathmins are positive regulators for neuronal differentiation. SCG10 is a neuronal differentiation marker for nerve growth factor (NGF)-induced cell differentiation in phenochromocytoma cells (PC12 cells)^32^. The expressions of SCLIP and RB3 are also notably increased^33^,^34^. STMN4 is expressed consistently at a high level during differentiation in multiple neuroblastoma cell lines^35^. In addition, proper exogenous expressions of stathmins promote not only neurite extension in rat hippocampal primary cultured neuron cells^36^,^37^ but also dendritic arborization in purkinje cells^38^. In spite of all described evidences disclosing the vital role of stathmins in neuron differentiation, we still assert that Stmn4 has a previously undescribed role as a neurogenesis blocker in the developing zebrafish dorsal midbrain. The discrepancy may be due to the facts that mainly adult neurons or somatic cell lines were used in previous studies. Those cells are derived from terminally differentiated cells. In contrast, we used a developing brain in an *in vivo* context. In addition, what we based on is the expression of earliest neuron differentiation marker Elavl3 which represents a much earlier signal comparing to the previous morphological observation in neurite formation in culture^39^. So what we found may represent a regulatory signal leading to the initiation of neurogenesis in dorsal midbrain.

To investigate how Stmn4 regulate the initiation of neurogenesis, we observed that proliferating cells labeled by BrdU or pH3 signals diminish significantly in *stmn4*-deficient embryos. Indeed, a strong correlation between stathmin expression and cellular proliferation had already been revealed in previous reports. First, high correlation between these two was confirmed in both normal and malignant cells^40^,^41^. Next, both overexpression and inhibition of stathmin expression give rise to mitotic arrest^42^. This is not surprising since mitotic spindles which are essential for chromosome aggregation and cell division should be carefully mediated by microtubule associated proteins such as stathmins. Since stathmin is crucial for mitosis progression, the lengthening of G_2_ phase in *stmn4*-deficient embryos observed in this study might be due to the difficulty of forming mitotic spindle for entering mitosis. Interestingly, we further observed that the *stmn4* expression level in neuron progenitor cells should be properly controlled within a narrow window that less or higher expression of *stmn4* both resulted in premature differentiation in dorsal midbrain. Similarly, neurite formation was also reported to be inhibited by knocking down SCG10 or SCLIP and enhanced by overexpressing a proper but not overdose of SCG10 or SCLIP in rat hippocampal primary cultured neuron cells^36^,^37^. For this dynamic instability, the abrupt switching behavior between microtubule growth and shrinkage, may provide some clues. Silva and Cassimeris proposed that stathmin may stand as a gatekeeper at the G_2_/M checkpoint via regulation of Plk1 and Aurora A activities which act upstream of Cdc25a phosphatase to facilitate cell cycle progression by withdrawing inhibitory phosphrylations from CDK1 partially through microtubule-dependent mechanisms in Hela cells^43^. This is in accord with our findings that Cdc25a could rescue the precocious differentiation phenotypes in *stmn4* morphants. Interestingly, normal Plk1 activity requires stathmin expression within certain limits as well. It suggests that the homeostasis of Stmn4 within neuronal progenitor cells should be strictly controlled for their maintenance of cell cycle progression.

Boekhoorn *et al*.^14^ proposed that stathmin is involved in the maintenance of neural precursor cell types in the adult hippocampus^14^. In stathmin-knockout mice, the impaired cell proliferation leads to reduced number of nestin-positive or PSA-NCAM-positive proliferating cells accompanying with the increasing DCX-labeled differentiated neuron cells. Although further detailed characterization of neuronal lineages within dorsal midbrain still needs to be done, this strongly supports to our study that Stmn4 controls the neuronal cell fate through mediating Cdc25a particularly during development. The “Cell cycle length hypothesis” has been proposed and established to provide an explanation that cell cycle regulators could elicit effect on cell fate determination factors inversely^10^. Inhibitors of cdk/cyclin complex such as p21 or p27 are precisely regulated to lengthen G_1_ phase during development^44^ that allows proneural genes like *neurogenin2* (Ngn2) to be transcribed and accumulated above thresholds to promote differentiation^45^. Furthermore, the lengthening of G_1_ phase not only avoids two compatible events regarding mitosis and morphogenesis^46^, but permit changes of epigenetic marks and nuclear architecture to determine cell fates^47^,^48^. While many researches were emphasized on the regulation of G_1_ phase, a few studies have begun to shed light on the G_2_ phase regulation lately^49^. In chordates, a longer G_2_ phase, which was introduced into epidermal cells flanking the neural plate, was found to be necessary for neural tube closure^50^. Restricted *cdc25a* expression was also proved to facilitate extension of body length and differentiation of muscle lineage cells in zebrafish^51^. Taken together, our results revealed that a short G_2_ phase, maintained by a proper level of Stmn4 expression in dorsal midbrain to guarantee active mitosis in neural progenitor cell pools. Despite that further investigation on cell cycle regulation (e.g., prolonged G_2_ phase may contribute to shorten G_1_ phase inversely^27^) still needs to be accomplished. We have offered strong support to the determination of cell fate via G_2_/M transition in addition to the well-studied G_0_/G_1_ regulation.

Several signaling pathways have been well investigated to play vital roles in midbrain development. Among this, the isthmus, which secretes morphogens such as Wnts and Fgfs, elicits effects on proliferation and neuron specification majorly through regulating Her5 activity^9^. A previous model stated that Wnt has a bi-modal role in activating FGF activity or impeding FGF activity via the induction of Sprouty genes which are known FGF inhibitors^8^. Combinations of positive- and negative-feedbacks loops ignite a retrograde FGF regulation on Her5 which preserves the isthmus as a neuron-free region. Our findings showing negative regulation of *stmn4* expression upon inhibition of Wnt activity further supports that Stmn4 can be an alternative mitogenic factor downstream of the Wnt signaling pathway. Despite of the contradictory facts that merely STMN2 is a novel target of β-catenin/TCF signaling in hepatoma cells^52^, we found several potential TCF-bindings sites within the *stmn4* promoter (data not shown) also implies that Wnts directly regulate *stmn4* at the transcriptional level.

**Figure 8:**
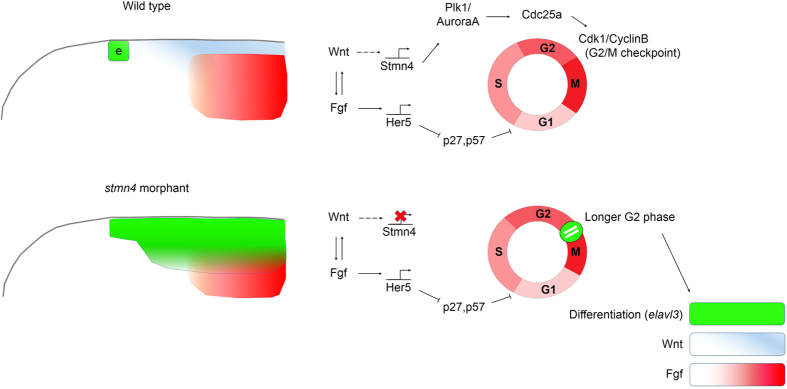
A proposed model describing interaction between proliferation and differentiation through Wnt-*Stmn4* regulation during early neurogenesis. At the neural keel stage, reciprocal interactions between Wnts (blue) and Fgfs (red) gradients underline the neuron-free intervening zone to trigger the expression of *her5*. Her5 then halts neurogenesis via inhibiting proneural genes and down-regulating p27 and p57, which hinder the progression of G_1_ phase through inactivating the Cdk/CyclinD complex. In addition, here we further propose that the Wnt signaling could keep the dorsal midbrain in a progenitor cell status via the induction of *stmn4* expression to mediate the progression of G_2_ phase by activating Cdc25a. In contrast, the *stmn4*-deficient embryos have a lower Cdc25a activity that leads to a longer G_2_ phase and results in precocious neuronal differentiation (green) in the dorsal midbrain.

In sum, we propose a model as shown in Fig. 8 that during the neural keel stage, Wnts could regulate the expression of *stmn4* in dorsal midbrain to control the length of G_2_ phase possibly via the coordination of G_2_/M checkpoint machinery. Deficiency or excess of Stmn4 would both lead to the retention of G_2_ phase that may allow accumulation of proneural gene transcripts to direct premature neuronal differentiation. Interestingly, *zic2a* and *zic5*, the proven targets of Wnt signaling pathway, display similar expression pattern in dorsal midbrain as *stmn4* and regulate not only mitosis but also dorsolateral hinge-point (DLHP) formation^53^. Moreover, the late onset of proliferation defect (16–18 ss; neural rod stage) shown in the *zic* morphants indicates Stmn4 may compensate the loss of Zic proteins for assisting G_2_ phase during neural keel stage or they have complementary mitogenic role between neural and neural rod/neural tube stage. Last, while most cell cycle regulators participating in midbrain development situate in the G_1_ phase, e.g. p27^Xic1^ regulated by Her5 through FGF activity could inhibit cyclinD/Cdk complex or Wnt could directly regulate *cyclin D1* and *n-myc* on transcription level, alternatively Stmn4 and Zic proteins (regulation of *cyclin B1*) influence the cell cycle progression via regulating the G_2_ phase. Regarding the rising evidences introducing G_2_ length control on cell fate determination, it will be fascinating to study how the regulation of G_1_ and/or G_2_ phases will cooperate spatially or temporally within the same platform, in particular midbrain development.

## Methods

### Ethics Statement

All experimental procedures on zebrafish were approved by the use of laboratory animal committee at National Taiwan University, Taipei, Taiwan (IACUC Approval ID: 103 Animal Use document No. 102) and carried out in accordance with the approved guidelines.

### Zebrafish

Wild type AB zebrafish (*Danio rerio*) (from Taiwan Zebrafish Core Facility at National Health Research Center, Taiwan), *Tg(HuC:Kaede)* fish (from Dr. Hitoshi Okamoto) and *Tg(hsp70l:dkk1-GFP)*^*w32*^ fish (from Taiwan Zebrafish Core Facility at Academia Sinica, Taiwan) were maintained at 28.5 °C on a 14-h light/10-h dark cycle. Embryos collected from natural mating were cultured and staged according to Kimmel *et al*.^54^.

### Plasmid constructions and Site-directed mutagenesis

Full-length coding sequences of *stmn4* was cloned from zebrafish cDNAs into the pCS2+ vector with BamHI (New England Biolabs (NEB), Ipswich, Massachusetts) and XbaI (NEB) for ectopic expression. DNA fragment (~300 bp) containing both MO binding sites was inserted in-frame with XLT-GFP in pCS2+ vector for MO efficiency check. Mutagenic primer pairs designed with desired mutations were used to generate the mutated plasmids by extending the original vectors with incorporating mutations, described as QuikChange™ Site-Directed Mutagenesis Kit protocol. We established three mutations in the serine sites of the SLD, including S59A, S71A and S81A, and also one combined mutations residing in the N’-terminal region, C20, 22A. For visualization, mCherry without stop codon were subcloned in frame with the *stmn4* gene in the pCS2+ vector with BamHI. For tagging Stmn4 directly, we cloned previous *stmn4* with combinatorial mutations of serine residues into pET23a (Novagen, Merck Milipore) with BamHI and XhoI (NEB), possessing 6X His on C’ terminal end. The two component induction system was first generated by adding novel viral 2A peptide sequence, from rhinitis A virus (PTV1); referred to P2A, residing within primers designed for the insertion of *stmn4* with 6X His tag into pT2MUASMCS^22^ with SalI (NEB) and EcoRI (NEB). The mCherry fused to the C-terminus of zebrafish histone variant H2A.F/Z from pME-H2AmCherry^55^ was further inserted into the N-terminal end of P2A with BglII (NEB) and SalI. The final constructs serves a convenient tool for conditional induction system when combining with Gal4 modules, with two translation-independent components which is easy to modify with several restriction sites to be selected. The shRNA^*stmn4_614*^ oligo (AAGACGTGAATGGTCACTTAC), designed against *stmn4* 3′ UTR by using the siRNA design tool available on GeneScript website, was generated through a two-step PCR with two overlapping primers forming BbsI restriction sites on both ends for cloning into miR30-based vector (SG1164) with CMV promoter (kind gift from Dr. Guo Su). This shRNA oligo could further shifted into UAS-based vectors (SG1180) with AccIII (NEB) and SnaBI^23^ (NEB).

### Cell transfection and co-localization analysis

Plasmids at 0.5 μg were used for Hela cell transfection. To label Golgi apparatus, we co-transfected cells with pEYFP-Golgi (Clonetech, TAKARA BIO Inc., Shiga, Japan). Images were collected using an LSM 780 confocal laser-scanning microscope (Carl Zeiss, Oberkochen, Germany). Co-localization was analyzed for each individual cell and quantified by the Fiji (formerly ImageJ) software with Pearson’s coefficient^56^.

### Embryo microinjections

Antisense morpholinos designed against *stmn4* (tMO_1_: GTCTCGATATGCTGCCAAGGTCATG; tMO_2_: AGTTAAGAAGTCTTCACTCTGTCCT), *p53* (tMO: 5′-GCGCCATTGCTTTGCAAGAATTG-3′) and random control MO (Random MO: NNNNNNNNNNNNNNNNNNNNNNNNN) were synthesized with a random based mixture at every position by Gene Tools. Plasmids for injection were prepared using the QIAGEN Plasmid Mini Kit (QIAGEN). Except for the corresponding dosages describing in the text, the doses we used in the conditional expression system were both 25 pg of pT2KhspGFF^22^ and pT2MUAS_H2AmCherry-2A-STMN4_3A or SG1180_STMN4 shRNA_614. All reagents were injected at the 1–2 cell stage.

### Generation of *stmn4* knockout lines by CRISPR/Cas9 system

Three guide RNAs (gRNAs, see sequences at Supplementary Table S1) were designed by the CHOPCHOP^57^ to target *stmn4* gene. Preparation of gRNAs includes annealing of two oligonucleotides, fill-in of single strand DNA overhangs with T4 DNA polymerase (NEB) and *in vitro* transcription by Megascript kits (Thermo Fisher Scientific, Waltham, Massachusetts)^58^. Cas9 mRNA was transcribed from the linearized pCS2-Cas9 (a kind gift from Dr. Alex Schier) using SP6 mMessage mMchaine kits (Ambion). Approximately 300 pg Cas9 mRNA and 25 pg gRNA were co-injected into 1- to 2- cell stage wildtype embryos and raised up to adults (F0). To identify carrier fish with sense mutations, F0 fish were crossed with wildtype fish to obtain F1 offspring. Genomic DNAs of founder fish were acquired by incubation of tail-fin clip with lysis buffer (proteinase K (0.4 mg/ml) diluted in TE buffer) at 55 °C for 2 h, inactivated at 85 °C for 15 min and then purified to be PCR templates. PCR products amplified by F1 and R1 primer pairs (see sequences at Supplementary Table S2) were cloned by TA cloning and subjected to Sanger sequencing to identify potential alleles. At least five batches of offspring embryos from different founders were then raised up to adulthood and identified for allele characterization by sequencing. Heterozygous F1 adults with same allele were inbreed to obtain the F2 generation, which were further genotyped with digesting purified PCR fragments amplified by F2 and R2 primer pairs (see sequences at Supplementary Table S2) from fin-clip genomic DNAs by BfaI restriction enzyme. Maternal zygotic mutant embryos (F3) from in-cross of homozygous mutant fish (F2) were utilized for further experiments.

### Whole-mount *in situ* hybridization and immunohistochemistry

DNA fragments of *lef1* were cloned from zebrafish cDNAs by RT-PCR and subcloned into pGEMT-easy vectors for probe synthesis. *elavl3* was obtained from Chin-Hwa Hu (National Taiwan Ocean University, Taiwan). *stmn4* was used previously by Shih *et al*.^15^. Whole-mount *in situ* hybridization (WISH) was performed as described using digoxigenin (DIG)-labeled antisense RNA probe^59^. Double *in situ* hybridization with modifications^60^ was carried out utilizing Fluorescein (FITC) labeled *elavl3* probe developed with the chromogenic substrate FastRed (Roche Applied Science) and DIG-labeled *stmn4* probe developed with NBT/BCIP (Promega). Stained embryos were mounted in glycerol, observed under a Leica S8AP0 stereomicroscope (Leica Microsystems) and photographed using a Canon 7D DSLR camera (Canon). Whole-mount immunohistochemistry staining was conducted as previously described^61^ by using either Rabbit Kaede antibody (PM012M, MBL International corporation, 1:500), Rabbit 6X His antibody (GTX115045, Genetex, 1:500), mouse alpha tubulin antibody (T 9026, Sigma-Aldrich, Merck, 1:300) or rat mCherry antibody (M11217, Molecular probes, Thermo Fisher Scientific, 1:300). Goat Secondary antibodies were combined with primary antibodies respectively with goat anti-mouse or anti-rabbit IgG conjugated with Alexa Fluor 488 or Alexa Fluor 568 (Molecular probes, 1:500). Images were collected utilizing LSM 780 confocal laser-scanning microscope (Carl Zeiss).

### Cell proliferation and G_2_ phase length analyses

Cell proliferation and G_2_ phase length were analyzed by specific experimental design described previously (Fig. 6a). Embryos were first treated with 10 mM 5-bromo-2′-deoxyuridine (BrdU, Sigma-Aldrich) pulses as described^62^ at 16 hpf and then fixed with fresh 4% paraformaldehyde (PFA) in phosphate-buffered saline (PBS) every hour until 19 hpf. Double IHC was performed with mouse BrdU antibody (B2531, Sigma-Aldrich, 1:250) and rabbit P-H3 antibody (GTX128116, Genetex, 1:1000) to mark cells in S phase and in M phase with corresponding secondary antibodies (1:500). Images were collected utilizing LSM 780 confocal laser-scanning microscope (Carl Zeiss) with 20X lens scanning the dorsal midbrain. Every individual embryo was scanned reaching 25 μm deep from top (10 stacks, 2.5 μm per stack) and shown with represented *z* stack. Furthermore, G_2_ phase length was determined by the percentage of labeled mitosis (PLM) paradigm^28^ which reveal the progression of the BrdU and P-H3 co-labeled nuclei after BrdU incorporation. Due to its proportionality to G_2_ length, it is simple to predict the duration of G_2_ phase by 50% of co-labeled nuclei in average.

### Confocal microscopy imaging, time-lapse recording and data analysis

Except for the MO efficiency check, all the other fluorescence photos were taken by LSM 780 confocal laser-scanning microscope (Carl Zeiss). For general scanning of the dorsal midbrain, stacks composed of 10 layers with 2.5 μm for each were performed. For detailed investigation of microtubule dynamics (Fig. S3b–d), thinner fraction of slices (1 μm) were scanned. All the collected data were further processed by the Fiji software^56^. Plugins “Skeletonized” and “AnalyzeSkeleton” were utilized to transform and analyze the 3D structure of microtubule patterns generated by confocal microscopy^63^. From the diversified raw data, polymerized microtubules and depolymerized microtubules were defined by the amount of slab voxels with more than 100 voxels giving rise to polymerized ones and less than 10 voxels to be depolymerized ones. For time-lapse recording, all the data were scanned using FV10i-DOC confocal laser-scanning microscopy (Olympus). Every frame photos were stacked images of 10 layers with 2.5 μm for each and taken every 6 minutes until 16 hpf.

### Quantitative real-time PCR

RNA extracts from 18 hpf *Tg(hsp70l:dkk1-GFP)*^*w32*^ embryos or 1 dpf wildtype embryos co-injected with shRNA^*stmn4_614*^ and pT2KhspGFF were extracted by RNAzol (Molecular Research Center) and synthesized by M-MLV reverse transcriptase (Promega) with oligo-dT. Gene-specific primers were designed by Vector NTI (Invitrogen, Thermo Fisher Scientific). All the qPCR data were collected by Bio-Rad iQ5 (Bio-Rad) using QuatiFastSYBR Green PCR Master Mix (Qiagen).

### Statistical analysis

All experimental values are presented as mean ± standard error and were analyzed by one-way ANOVA. The number in bottom or above the bar indicates the total sample number in one experimental condition. Groups denoted with different lettering refer to statistical significance (p < 0.05).

## Additional Information

**How to cite this article**: Lin, M.-J. and Lee, S.-J. Stathmin-like 4 is critical for the maintenance of neural progenitor cells in dorsal midbrain of zebrafish larvae. *Sci. Rep*. **6**, 36188; doi: 10.1038/srep36188 (2016).

**Publisher’s note**: Springer Nature remains neutral with regard to jurisdictional claims in published maps and institutional affiliations.

## Supplementary Material

Supplementary Information

Supplementary Video S1

Supplementary Video S2
