# Supplementary material for: Stathmin-like 4 is critical for the maintenance of neural progenitor cells in dorsal midbrain of zebrafish larvae
Source: Sci Rep. 2016 Nov 7;6:36188. doi: 10.1038/srep36188 (PMC5098158; doi:10.1038/srep36188)
Supplement: Supplementary Information [file srep36188-s1.doc]

**Stathmin-like 4 is critical for the maintenance of neural progenitor cells in dorsal midbrain of zebrafish larvae**

Meng-Ju Lin^1^ and Shyh-Jye Lee^1,2,3,4*^

^1^Department of Life Science, National Taiwan University, Taipei, Taiwan. ^2^Research Center for Developmental Biology and Regenerative Medicine, National Taiwan University, Taipei, Taiwan. ^3^Center for Systems Biology, National Taiwan University, Taipei, Taiwan. ^4^Center for Biotechnology, National Taiwan University, Taipei, Taiwan.

**Supplementary Tables**

Table S1 List of gRNAs used.

|  | gRNA+PAM | Genomic location | Exon | Strand |
| --- | --- | --- | --- | --- |
| #1 | GAGCACAGAAGTGACACTAGAGG | ch20:34822438 | 3 | + |
| #2 | GATCCTACTTGAAGATGCGGTGG | ch20:34822268 | 4 | - |
| #3 | GGTGCAACATCAAAGGAAGGGGG | ch20:34822159 | 4 | + |

Table S2 List of primers used.

| Target gene | Forward | Reverse |
| --- | --- | --- |
| *stmn4* Ex.3-5 F1/R1 | CAGCATATCGAGACAAGGTGAAGGA | GCCTCCTGGAACTAGAGAAAACAGTACA |
| *stmn4* Ex.3-5 F2/R2 | CCAGCATCTTTTAAAGAGCGATCC | GTCAGCCTCCTGTGTTTATGATCAG |
| *stmn1b* | TGTGTGTAATCAGTTCTGTCT | GCTCAAAGGATGGCAGTA |
| *stmn2a* | ATCCTCATAAACTCCCAATTACAG | TCGCTTCACTTCCAGAGAT |
| *stmn3* | CCCAGACACTGGCACTGCCA | AGTTACCCTGCGCCTGGCCT |
| *stmn4* | CAAGTCCTGTCTCTTCACA | TAGCACTGACTGGAGGTT |
| *lef1* | TCCTACACCTGTCCCTAAGTTAT | TGTTACACCAATGTCTGTGAAGA |
| *ef1α* | TGCCTTCGTCCCAATTTCAG | TACCCTCCTTGCGCTCAATC |

**Supplementary Figures**

**
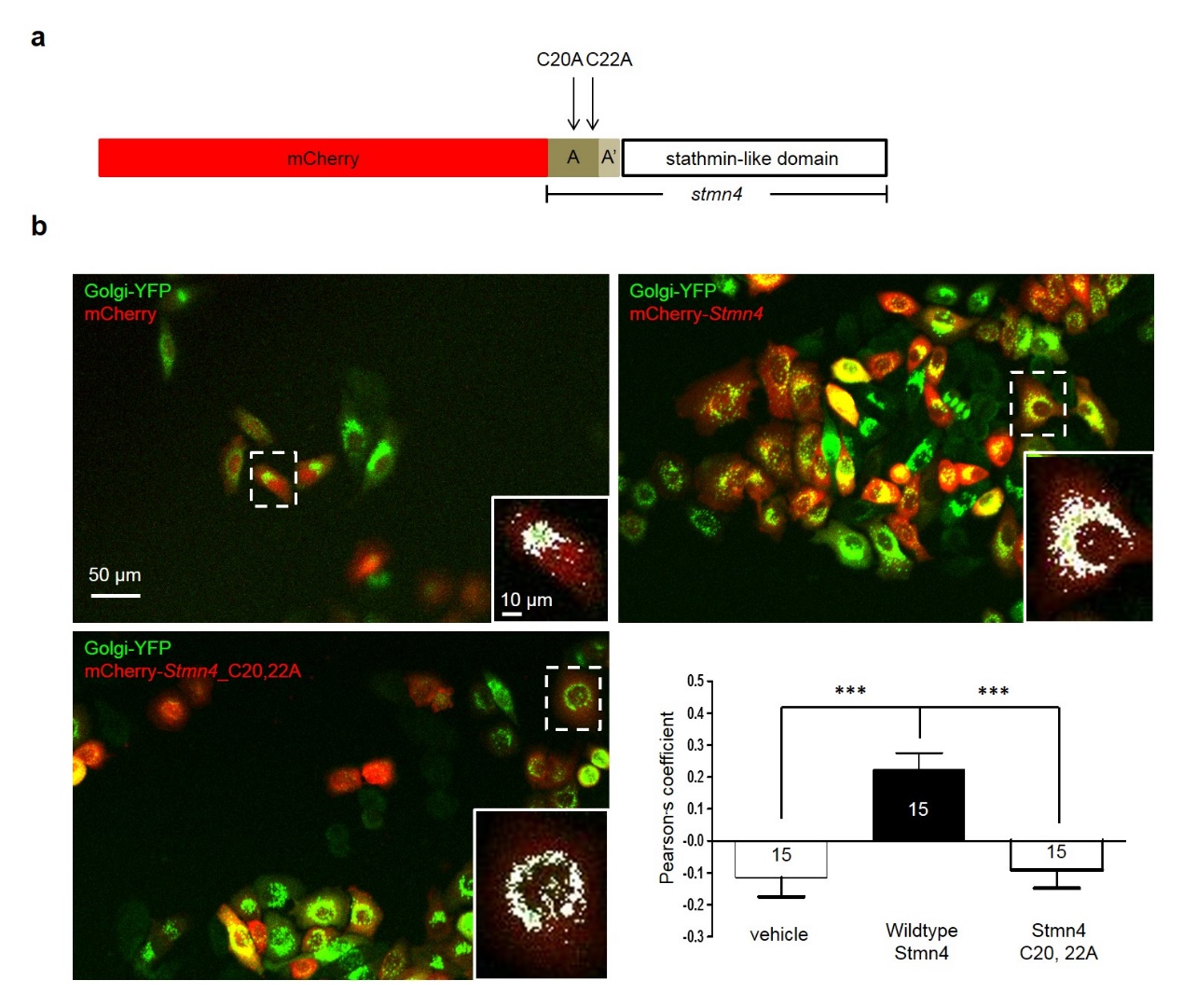
**

**Fig. S1. Zebrafish Stmn4 was localized to Golgi apparatus in a cysteine-dependent manner.** (**a**) Construct design: the mCherry with or without a C terminal full-length coding sequence of *stmn4* gene, containing two N terminal domains (A and A’) and a stathmin-like domain was cloned into the pCS2+ vector. Two cysteine sites, C20 and C22, on the A domain were changed to alanine that could interfere with Golgi membrane-tethering of *Stmn4*. (**b**) Hela cells were co-transfected with Golgi-YFP to reveal Golgi in green and mCherry containing pCS2+ vector without or with wildtype or Cysteine-mutated stathmin as indicated in red. Cells were imaged and photographed under confocal microscopy and the overlapping signal in yellow indicated the stathmin was located in Golgi apparatus. A higher magnification of a dashed area is shown at the lower right corner for each panel. Scale bars are shown only on the upper left panel. To analyze the localization of Stmn4 in Golgi apparatus, the overlapping yellow signals were changed to white for clarity using the ImageJ software as shown in the higher magnification graphs. The correlations between treatments for the Golgi localization of stathmin are shown by calculating their Pearson’s correlation coefficients and statistically analyzed by one-way ANOVA as resented in the lower right graph. Data represents mean + s.e.m. **P*<0.05.


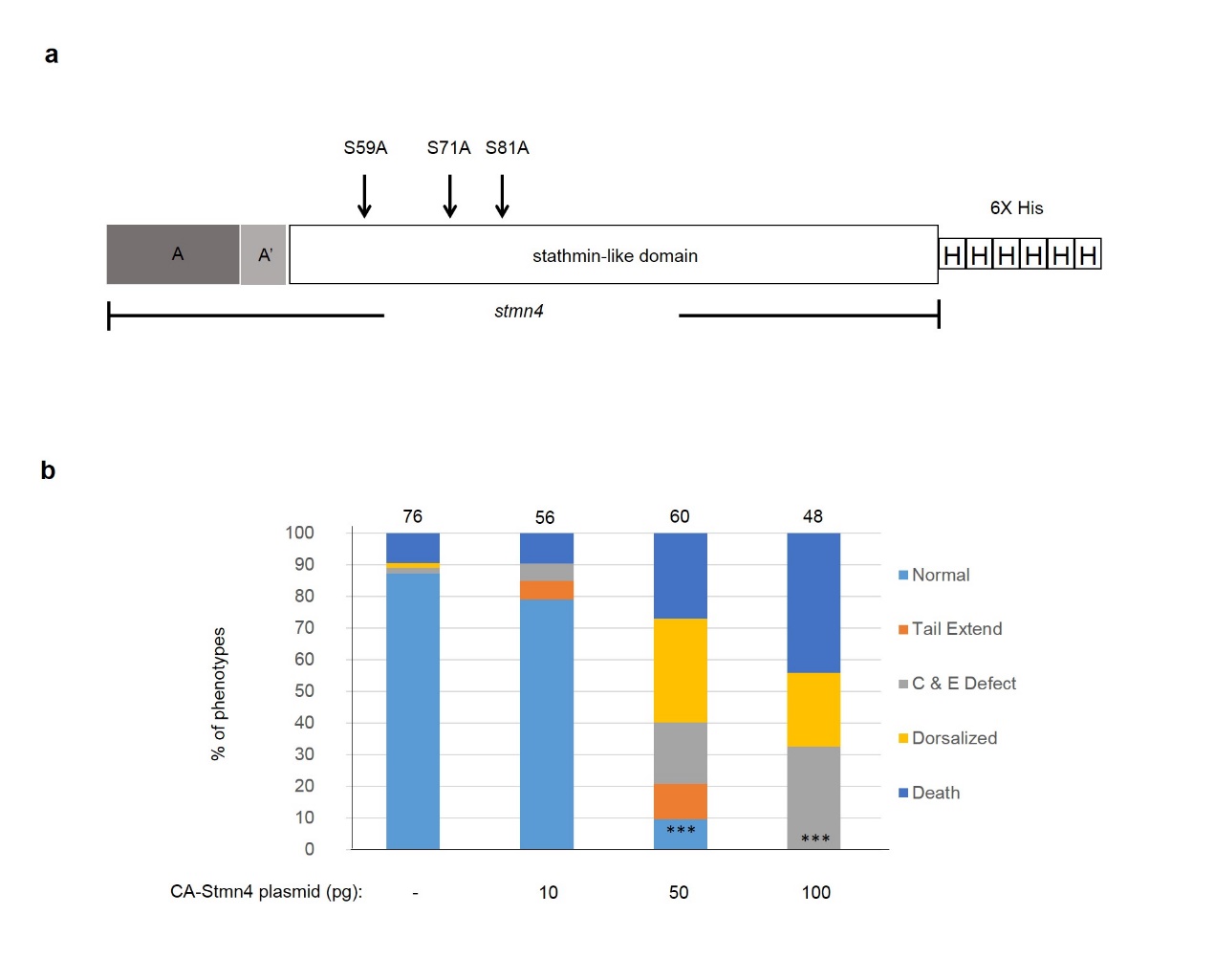


**Fig. S2. Ubiquitous expression of CA-Stmn4 disrupts patterning during gastrulation.** (**a**) Diagrams displaying the individual mutations of the conserved phosphorylated serine residues, S59A, S71A and S81A. (**b**) Overexpression of CA-Stmn4 construct destroying patterning during gastrulation lead to five features: normal, piggy tail, convergence-extension (CE) defect, dorsalized and death. Quantification data are presented here with different doses depicted below.


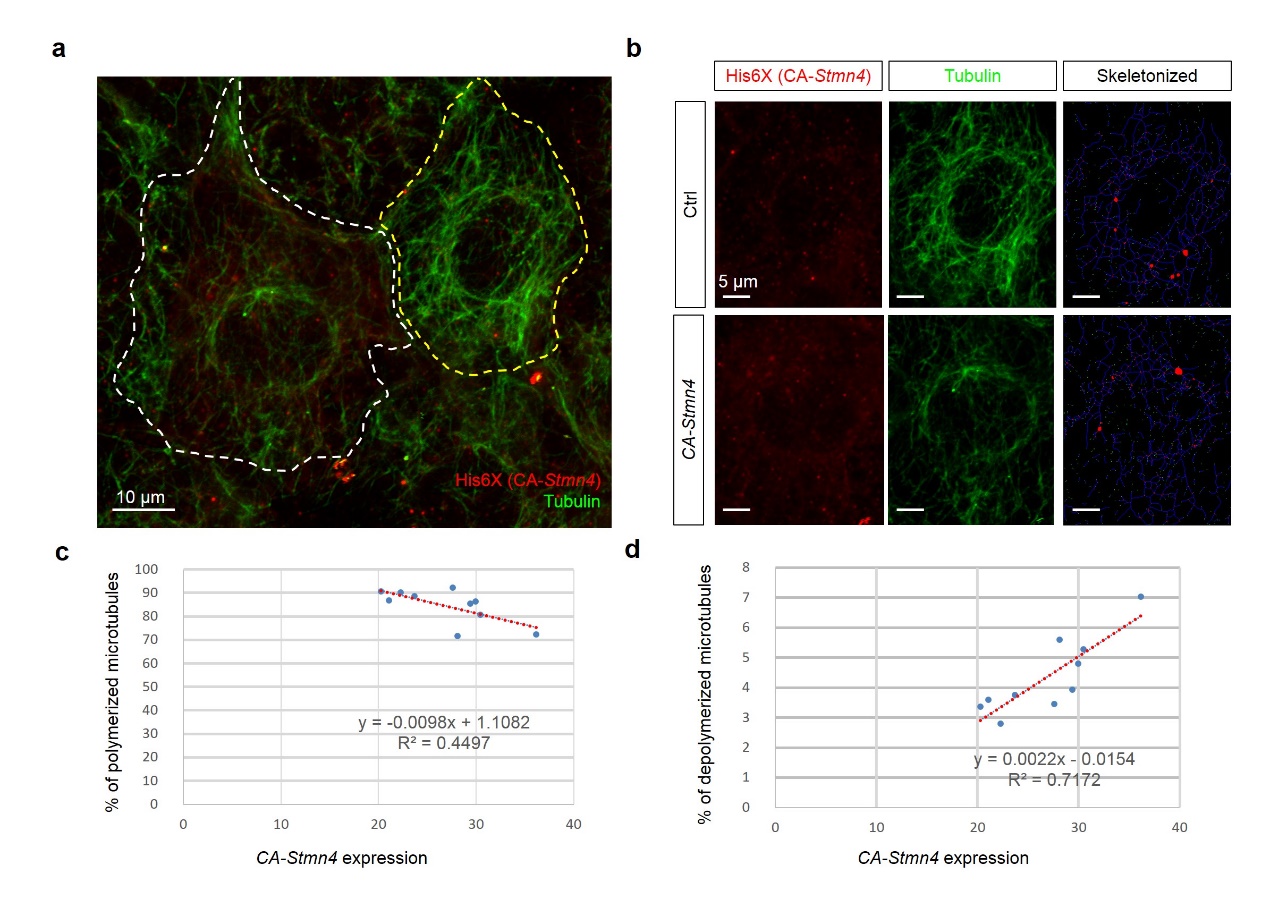


**Fig. S3. Overexpression of constitutively active *stmn4* induced depolymerization of microtubules in zebrafish embryos.** Zebrafish embryos were injected with plasmids containing without or with constitutively active Stmn4 (CA-Stmn4) as described in Fig. S2, cultured and fixed at the bud stage. The fixed embryos were subjected to immunohistochemistry against histidine and alpha-tubulin as described in the Materials and Methods. After staining and washing, embryos were imaged and photographed under confocal microscopy. (**a**) Representative image of cell overexpressed with *ca-stmn4* (labeled by α-His6X, red signals) co-staining tubulin (indicated by green signals). Scale bars equal 10 μm. (**b**) Two separate channels one showing differential CA-Stmn4 expression level, strong (white dashed polygon, lower) and weak (yellow dashed polygon, upper), and corresponding tubulin patterns. Plugins “Skeletonized” and “AnalyzeSkeleton” of ImageJ software were utilized to transform into the right panels with labeling slabs (blue), junctions (red) and ends (green). (**c**,**d**) Percentage of polymerized or depolymerized microtubules among total tubulins were analyzed.

**Video Legends**

Video 1. ***Neuronal differentiation in the dorsal midbrain of untreated embryos***

Few differentiated neurons (green, arrows) appeared in dorsal midbrain until 16 hpf in untreated embryos.

Video 2. ***Neuronal differentiation in the dorsal midbrain of ca-stmn4-expressing embryos***

Overexpression of *ca-stmn4* (red) induced premature neuronal differentiation (green, arrows) and apoptosis (arrowheads) in individual neuronal progenitor cells of dorsal midbrain.
